# Relaminarization of jet impingement on a flat plate using separation-induced transition correction turbulence modeling preliminarily applied in archeological applications

**DOI:** 10.1016/j.heliyon.2024.e26040

**Published:** 2024-02-08

**Authors:** Mongkol Kaewbumrung, Chalermpol Plengsa-Ard

**Affiliations:** a Department of Mechanical Engineering, Faculty of Engineering and Architecture, Rajamangala University of Technology Suvarnabhumi, Phranakhon Si Ayutthaya, 13000, Thailand; b Department of Mechanical Engineering, Faculty of Engineering, Kasetsart University, Bangkok, Thailand

**Keywords:** Relaminarization, Reverse transition, Ancient mural painting, Archeological application

## Abstract

The focus of the study was the validation of transition flow model which works with jet impingement applications. Reynolds averaged Navier Stokes (RANS) techniques, including a variation of transition options, such as γ−GEKO−k−ω, γ−SST−k−ω, transition SST and transition k−kl−ω, were employed to simulate the turbulent flow fields. ANSYS-Fluent 2022R2 was the tool used in the study and a constant surface heat flux on a flat plate was chosen for the test case. The flow characteristics and averaged Nusselt number prediction were investigated and compared with measurement data. The results show that the collapse of turbulence performed the flow became lamina-like in the transition region, 1 < r/D < 3, while the shape of velocity distribution within that region was parabolic profiles near a target wall. That means the relaminarization mechanism was captured by the transition SST model. Moreover, the thermal results demonstrated that the highest values of the Nusselt number prediction were located near the stagnation point and decreased monotonically within the wall jet regions. The predicted results from γ−SSTk−ω and transition SST models provided strong agreement with experimental data. Secondary peaks of the Nusselt number in the radial direction were also depicted by transition SST models, and these second peaks were still aligned on the ratio (1 ≤ r/D ≤ 3) which was not impacted by jet diameter variation. This range will be used in the next step of developing a prototype of a dehumidifying unit for use in archeological applications.

## 1 Introduction

Relaminarization or the reverse transition of turbulent flows has been observed in various science and engineering applications [1]; however, the accurate prediction of these flow patterns remains a complicated matter. These flow mechanisms can be analyzed with some of the explanatory flow visualization techniques compared to using numerical simulation [2]. From research on wall-bounded flows, bursting phenomena are identified as a primary source of the turbulence mechanism that reverses the generation of Reynolds stress and turbulence energy near wall regions [3]. Recently, leapfrog development in high-performance computations has been developed for direct numerical simulations (DNS) to provide a substantial valuable scientific database of the bursting phenomena and shows that quasi-coherent streamwise vortices dominate the bursting phenomena and sustain turbulence near the wall [4,5]. Hence, to better understand relaminarization, it is necessary to focus on the behavior of the streamwise vortices mechanism, the critical Reynolds number, and the intermittency of the flow structure related to the friction coefficient in terms of the Nusselt number.

In literature reviews, the reverse transition from turbulent to laminar flow has been studied by many investigators [6,7]. From the beginning of biomedical applications, relaminarization was investigated by Tabe et al. [8]. The numerical results were in excellent agreement with the experimental data using the SSTk−ω transitional model. Turbulent transition involved with complex fluid structures was evaluated based on several flow criteria. Kelly et al. [9] investigated the influence of shear-thinning blood rheology to produce biomedical information about cardiovascular diseases. The comprehensive numerical simulation results suggested that shear-thinning and Newtonian rheologies delay transition behavior. Ding et al. [10] studied the transitional pulsatile flow using stenosises. They found that the flows for stenosises had laminar characteristics and became turbulent through shear-layer instability under a strong adverse pressure gradient which was responsible for cellular dysfunction, leading to the development of atherosclerotic lesions [11,12]. The initial relaminarization of engineering applications was analyzed by Roback et al. [13], who proposed a useful application of computational fluid dynamics capability to predict relaminarization. The dilation effect in relaminarization of a supersonic turbulent boundary layer was studied by Teramoto et al. [14]. Later, Currao et al. [15] extended the deep insight into the relaminarization mechanism for estimating the magnitude and location of peak heat-flux before adopting it in the thermal protection systems of hypersonic vehicles. The reformulation of the sustaining turbulence technique was fundamentally purposed by Krumbein et al. [16] and applied in the NASA common research model for natural laminar flow configuration. Then, Li et al. [17] studied the transition flow on an airfoil as influenced by surface roughness. They concluded that the frequency, transition, and transition location were affected by the roughness. To predict boundary layer relaminarization, the laminar separation bubble over a supercritical airfoil was investigated numerically and experimentally by Tatar et al. [18]. The transition points were observed using the second derivative of surface pressure data, hot-film measurements, and numerical transition models. Issaev et al. [19] observed three distinctly different mixing regimes that were separated by transitional values of turbulence along with the parameterization schemes. The fundamental flow physics associated with a delay of transition and relaminarization was numerically demonstrated by Hosseinverdi et al. [20]. In high fidelity numerical simulation, DNS of the polymer-induced flow relaminarization and drag enhancement in a spanwise-rotating plane couette flow was deliberated by Zhu et al. [21]. Applying these complex calculations showed that viscoelastic flow transition arises gradually by weakening, resulting from a decrease in small-scale vortices. Kühnen et al. [22] in their investigation into the temporal relaminarization mechanism, showed that modification of the streamwise velocity profiles in pipe flows significantly affected the local skin friction, drag reduction, and weakening of near-wall turbulent production cycles. These results were consistent with the findings of Wang and Gharib [23], who applied a dynamic free-slip boundary to investigate the local relaminarization mechanism induced by a dynamic free-slip boundary and the Weissenberg number. Furthermore, the spanwise-rotation-driven flow transitions in the viscoelastic plane were investigated by Zhu et al. [24].

Because the complex flow mechanism of re-transition, separation, and relaminarization is still open to speculation, scientists and engineers may develop and produce new theories to predict accurate flow patterns with minimized computational resources. DNS is the best choice for solving all turbulence scales following the Kolmogorov approach but consumes extremely high computational resources and is not feasible under realistic industry practice. Consequently, a new turbulence model is needed that can minimize computational expense [25,26]. From these limitations, Cerbus [27] refined the mechanism of laminar and turbulent flow using Prandtl-Tietjens intermittency to explain the fluctuations in internal flow resistance. De Santis et al. [28] enhanced the turbulence in laminar separation bubbles, with the results from the new formulation agreeing well with available experimental data. Carnes and Coder [29] analyzed the local-correlation laminar-turbulent transition model to develop a new understanding of near-wall behavior. They proposed a new turbulence index function to detect the laminar-turbulent transition. Usually, laminar separation bubbles are one of the critical aspects of engineering; in the laminar regime, with turbulence developing inside the recirculation region enhancing momentum transport, so the flow can reattach. Samson and Sarkar [30] initialized primary experimental investigation of a laminar separation bubble on the leading edge under Reynolds number variation to elaborate on the flow structure and visualization detail and concluded that the laminar separation bubble length was highly dependent on the Reynolds number. Lopez and Walters [31] applied the kinetic energy approach to develop a new eddy-viscosity turbulence model to predict the laminar, transitional, and turbulent flow processes. Zhang et al. [32] explored the effect from a period of gust inflow and found that flow separation occurred inside the curved section. Hajaali and Stoesser [33] proposed a new concept to investigate the flow separation dynamics in 3D asymmetric diffusers by analyzing the instantaneous backflow, power density spectra, and reduction phases. Hattori et al. [34] described an experimental study of unforced laminar-to-turbulent transition in pipe flow. The visual observations revealed that the flow structure depended on variation in the Reynolds numbers corresponding with the peak powers and an experiment involving impinging a water jet was confirmed by works of Joppa et al. [35].

Additionally, it is not easy to understand the flow mechanism in any field with general turbulence models [36,37], as well as requiring extremely large amounts of computational resources that are not practical in an industrial application or for those having restricted computational resources [38,39]. To overcome these limits, the objective of the current paper was to validate new turbulence models, including separation-induced transition corrections such as γ−GEKOk−ω, γ−SSTk−ω, transition SST and transition k−kl−ω, which are included in a commercial software package ANSYS-Fluent. The validation methodology used the standard academic test case. The primary investigation variables were Nusselt number, velocity profile, and turbulent kinetic energy compared with previous experimental data [40,41]. After obtaining all the high-accuracy flow characteristics, these will be preliminarily applied to ancient mural painting restoration and cleaning. The application focused on the numerical simulation of jet impingement on a mural wall, utilizing a turbulence model that incorporates a correction for separation-induced transition. Although the previous research $v^{2}-f$ turbulence model has superior results compared with Reynolds stress turbulence models, $v^{2}-f$ is not available in ANSYS 2022R2 [46]; consequently there is a need for the validation and verification of a turbulence model of jet impingement to capture the relaminarization with an accurate second peak of the Nusselt number position. The utilization of this methodology in the field of archeology is not well-established in the literature, as it requires a substantial level of cross-disciplinary integration between the fields of engineering, mathematics, and archeology. The integration of these areas is challenging; however, this simulation has the potential to provide valuable insights into the field of archeology.

## 2 Mathematical formulation

**Fig. 1:**
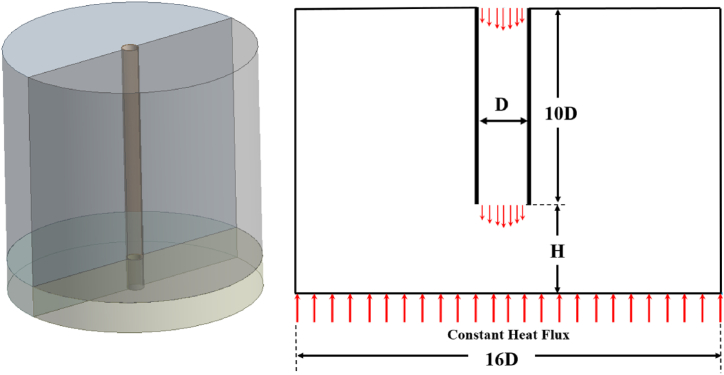
Computational domain of jet impingement.

Jet impingements on heated walls were investigated, while air was selected with a Newtonian fluid assumption. The initial flow parameter in a long pipe with a thin wall was a nozzle with a diameter D = 26.5 mm. The normal distance from the nozzle exit to a wall surface was fixed at H/D = 2 and circular base plate dimensions were about 16D x 16D which was far enough from any outlet pressure effect. The computational domain schematic for this problem is provided in Fig. 1, with the vertical axis x and the horizontal reference axis r included. Cylindrical coordinates are used for the computational domain of the impinging jet. The original point (r = 0) is located at the center line. The velocity components in the axial (x), radial (r) and azimuthal (θ) directions are denoted by U, W and V, respectively.

The present study employs an accurate and reliable laminar-turbulent transition model called $\gamma -{\text{Re}}_{\theta }$ model, the intermittency model was introduced in a transport equation with the intermittency function (γ) is defined as the fraction of time the flow remains turbulent coupled with the SSTk−ω model [43]. This function was applied to switch on the production term of the turbulent kinetic energy downstream of the separation-transition point. The extended formulation of the intermittency equation was improved to consider the rapid onset of transitions that are affected by the separation of the laminar boundary layer. Finally, the model was calibrated with the standard transition onset equation [44,45].

### 2.1 Transport equations for transition SST turbulence model

The SSTk−ω model was coupled with the intermittency, transition onset, and momentum-thickness Reynolds number. Langtry and Menter [47,48] proposed the transport equation for intermittency (γ) as Eq. (1):

(1) $$\frac{\partial \left(\rho \gamma \right)}{\partial t}+\frac{\partial \left(\rho U_{j}\gamma \right)}{\partial x_{j}}={ϒ}_{\gamma 1}-\Gamma_{\gamma 1}+{ϒ}_{\gamma 2}-\Gamma_{\gamma 2}+\frac{\partial }{\partial x_{j}}\left[\left(\mu +\frac{\mu_{t}}{\sigma_{\gamma }}\right)\frac{\partial \gamma }{\partial x_{j}}\right]$$

Both source terms for the transition and the destruction (relaminarization) are introduced by:

$$\begin{array}{l} {ϒ}_{\gamma 1}=2F_{\text{length}}\rho S{\left[\gamma F_{\text{onset}}\right]}^{0.5},\Gamma_{\gamma 1}=\gamma {ϒ}_{\gamma 1}\text{,}\\ {ϒ}_{\gamma 2}=0.06\rho \Omega \gamma F_{turb},\Gamma_{\gamma 2}=50\gamma {ϒ}_{\gamma 2} \end{array}$$

where *S* represents the strain rate magnitude, while F_length_ defines an empirical correlation that controls the length of the transition zones. The term Ω characterizes the magnitude of vorticity of flow fields. The set of equations below demonstrates the transition onsets as following Eqs. (2), (3), (4), (5), (6), (7), (8):

(2) $$F_{\text{onset}\;1}=\frac{{\text{Re}}_{V}}{2.193{\text{Re}}_{\theta_{C}}}$$

(3) $$F_{\text{onset}\;2}=\min\left(\max\left(F_{\text{onset}\;1},F_{\text{onset}\;1}^{4}\right),2.0\right)$$

(4) $$F_{\text{onset}\;3}=\max\left(1-{\left(\frac{R_{T}}{2.5}\right)}^{3},0\right)$$

(5) $$F_{\text{onset}\;}=\max\left(F_{\text{onset}\;2}-F_{\text{onset}\;3},0\right)$$

(6) $$F_{\text{turb}}=e^{{\left(-R_{T}/4\right)}^{4}}$$

(7) $${\text{Re}}_{V}=\frac{\rho y^{2}S}{\mu }$$

(8) $$R_{T}=\frac{\rho k}{\mu \omega }$$

To improve transition predictions, the transport equation for the momentum-thickness Reynolds number $\left({\text{Re}}_{\theta t}\right)$ is also shown by Eq. (9) from Menter et al. [47,48] as:

(9) $$\frac{\partial \left(\rho {\tilde{\text{Re}}}_{\theta t}\right)}{\partial t}+\frac{\partial \left(\rho U_{j}{\tilde{\text{Re}}}_{\theta t}\right)}{\partial x_{j}}=\Psi_{\theta t}+\frac{\partial }{\partial x_{j}}\left[2\left(\mu +\mu_{t}\right)\frac{\partial {\tilde{\text{Re}}}_{\theta t}}{\partial x_{j}}\right]$$

In the $\gamma -{\text{Re}}_{\theta }$ model, there are some parameters related to other parameters [49], such as the transition onset obtained from experiment data $\left(F_{onset}\right)$, the length of the transition zone $\left(F_{length}\right)$, and the critical Reynolds number $\left({\text{Re}}_{\theta_{c}}\right)$, that are defined by.

$${\text{Re}}_{\theta t}=f\left(Tu,\zeta \right),F_{length}=f\left({\tilde{\text{Re}}}_{\theta t}\right),{\text{Re}}_{\theta c}=f\left({\tilde{\text{Re}}}_{\theta t}\right)$$

As shown above, the first empirical parameter is dependent on the level of turbulence intensity (Tu) and the pressure gradient of Thwaites (ζ). This turbulence intensity term is obtained from $Tu=\left(100/U\right)\sqrt{\left(2/3\right)k}$, where k represents turbulent kinetic energy and U is local freestream velocity. Additionally, one of the source terms from the above transport equation is written as following Eqs. (10), (11), (12), (13), (14), (15), (16):

(10) $$\Psi_{\theta t}=0.03\frac{\rho^{2}U^{2}}{500\mu }\left({\text{Re}}_{\theta t}-{\tilde{\text{Re}}}_{\theta t}\right)\left(1.0-F_{\theta t}\right)$$

(11) $$F_{\theta t}=\min\left(\max\left(F_{\text{wake}}e^{{\left(-y/\delta \right)}^{4}},1.0-{\left(\frac{\gamma -1/50}{1.0-1/50}\right)}^{2}\right),1.0\right)$$

(12) $$\delta =\frac{50\Omega y}{U}\delta_{BL}$$

(13) $$\delta_{BL}=7.5\theta_{BL}$$

(14) $$\theta_{BL}=\frac{{\tilde{\text{Re}}}_{\theta t}\mu }{\rho U}$$

(15) $$F_{\text{wake}}=e^{-{\left({\text{Re}}_{\omega }/1e5\right)}^{2}}$$

(16) $${\text{Re}}_{\omega }=\frac{\rho \omega y^{2}}{\mu }$$

For the boundary condition of ${\tilde{\text{Re}}}_{\theta t}$, the zero normal flux boundary condition near walls is chosen. At the inlet surface, the initial condition for ${\tilde{\text{Re}}}_{\theta t}$ is determined from the information of correlations which relates to the inlet turbulence intensity calculation. More information is provided in the literature [47,48].

### 2.2 Separation-induced transition correction

The modified separation-induced transition is defined by Eqs. (17), (18):

(17) $$\gamma_{\text{sep}}=\min\left(2\;\max\left[\left(\frac{{\text{Re}}_{V}}{3.235{\text{Re}}_{\theta_{c}}}\right)-1,0\right]e^{-{\left(R_{T}/20\right)}^{4}},2\right)F_{\theta t}$$

(18) $$\gamma_{eff}=\max\left(\gamma ,\gamma_{sep}\right)$$

To improve the accuracy of separated flow predictions in transition regions, the model coefficient, which enforces the relationship between ${\text{Re}}_{V}$ and ${\text{Re}}_{\theta c}$, was adjusted from 2.193 to 3.235 [47,48], the value at separation points where the shape factor is 3.5. The zero normal flux at a wall for the boundary condition was assumed and the initial condition of γ was set to 1.0 at the inlet.

### 2.3 Coupling transition model and SST transport equations

Only the transport k-equation was modified to make the transition options couple with the SST turbulence model, as shown in Eq. (19):

(19) $$\frac{\partial }{\partial t}\left(\rho k\right)+\frac{\partial }{\partial x_{i}}\left(\rho ku_{i}\right)=\frac{\partial }{\partial x_{j}}\left(\beta_{k}\frac{\partial k}{\partial x_{j}}\right)+\gamma_{eff}{\tilde{G}}_{k}-\min\left(\max\left(\gamma_{eff},0.1\right),1.0\right)Y_{k}$$

The terms ${\tilde{G}}_{k}$ and $Y_{k}$ can be considered as the original production and destruction terms, respectively. However, the production terms of the ω-equation remain unchanged. More detail can be found in the works of Menter et al. [47,48].

## 3 Solution method

To match with experimental conditions, a downward directed jet impinging on a flat wall with Reynolds number = 23,000 was set to match the Reynold number used in the experiment. More details of experimental set up can be found in the literature [40]. The internal velocity profiles were solved in a long pipe until fully developed with an average air velocity of 15.54 m/s and an air density of 1 kg/m^3^. Incompressible flow assumptions were applied for this study due to low Mach number conditions. The pipe wall boundary conditions were defined as smooth adiabatic walls. The inlet and initial condition of air temperature in the pipe was set to 298 K. A constant heat flux of 300 W/m^2^ on the target plate was set for thermal boundary conditions, while the current studies used the outlet pressure boundary conditions at exit. The steady state simulation was demonstrated using ANSYS-Fluent 2022R2. There were 5.4 million total computational fluid cells, and the CPU time was about 24 h with 32 cores. The following equation as shown in Eq. (20) introduces the average Nusselt number prediction:

(20) $$Nu=\frac{q_{w}D}{\left(T-T_{ref}\right)\Phi }$$

where D is the exit air jet diameter, Φ represents the thermal conductivity of the fluids, T is the local surface temperature and $q_{w}$ is the local convective heat flux. The reference temperature $T_{ref}$ is justified by using the air jet temperature at pipe exit.

### 3.1 Numerical setting

**Fig. 2:**
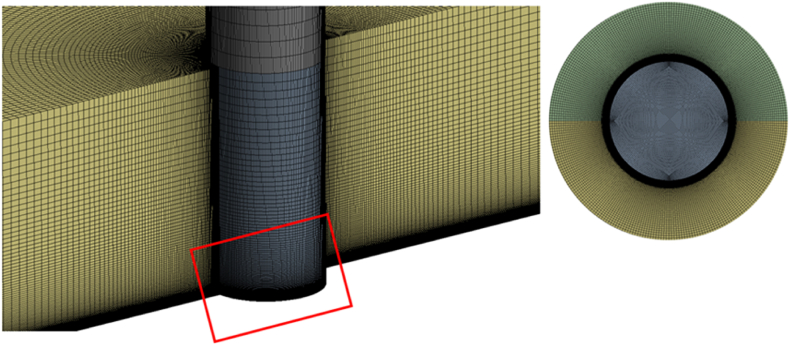
Computational grids (left) with special treatments for grids in a red box (right). (For interpretation of the references to colour in this figure legend, the reader is referred to the Web version of this article.)

Flow field behavior was simulated using time average techniques simulations with γ−GEKO−k−ω, γ−SST−k−ω, transition SST, and transition k−kl−ω and compared with the exit experiment data [40,41]. A pressure-based coupled algorithm for pressure-velocity and a Green-Gauss cell-based scheme for gradients were adopted in this simulation. The first grid point $\left(y^{+}\right)$ from the wall was 0.04 (<1), which was adequate to resolve flow fields near the wall's boundary layer, as shown in Fig. 2. However, this research had very complex flow behavior due to the separation and transition mechanisms; hence, we adjusted the spatial discretization and multigrid cycle as shown in Table 1.

**Table 1:** Numerical settings.

Solution control	Method
Spatial discretization	
Pressure	Standard
Momentum	Second Order upwind
Turbulent kinetic energy	Second Order upwind
Intermittency	Second Order upwind
Momentum thickness Reynolds number	Second Order upwind
Energy	Second Order upwind
Multigrid	
Flow	F-cycle
Turbulent kinetic energy	Flexible
Specific dissipation rate	Flexible
Intermittency	Flexible
Momentum thickness Reynolds number	Flexible
Energy	F-cycle

### 3.2 Grid independence analysis

**Fig. 3:**
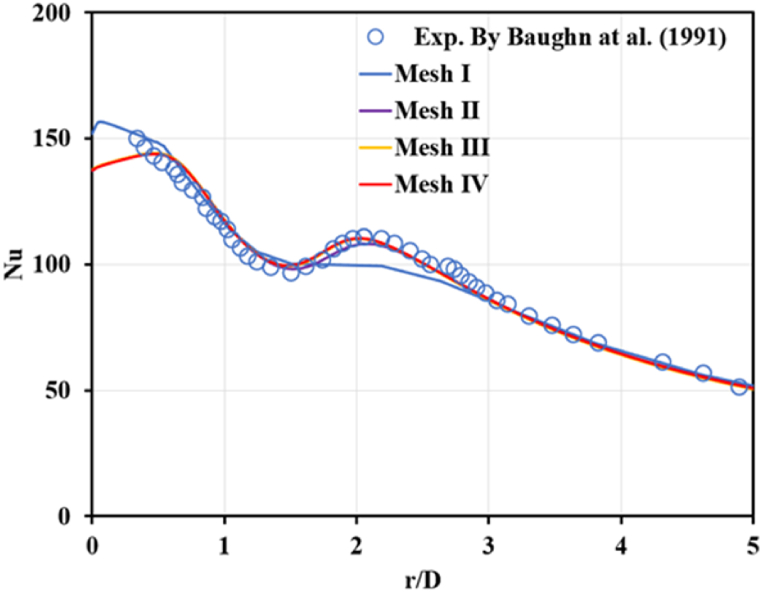
Results of grid independence analysis.

Time average technique simulations with transition SST models were used to simulate the flow field. We selected four cases with different numbers of grids to test based on the transition SST, as shown in Fig. 3. To minimize the error due to the grid size variation effect on the final solution, we selected Case Mesh IV for all simulation cases because it was in excellent agreement for all investigation ranges compared with published data [40]. Also, more detail of grid independence study can be found from the previous works by the same group of authors in the literature [42].

## 4 Results

This section consists of four parts. The first part investigates the effect of the general transition turbulent model, including the destruction/relaminarization and the modification of separation-induced transition sources, such as γ−GEKO−k−ω, γ−SST−k−ω, transition SST, and transition k−kl−ω, respectively, and the predicted results of the mean Nusselt number on a hot flat plate are shown. The available experimental data on the Nusselt number from Baughn et al. [40] are also plotted for comparison. The second part demonstrates the normalized mean jet velocity versus the axial vertical distance. The third part presents the mean turbulence kinetic energy profile to investigate the energy extracted from the mean flow. The final part presents the contour plots of Nusselt number and velocity profiles near walls in transition wall zones.

### 4.1 Effect of general turbulence model

**Fig. 4:**
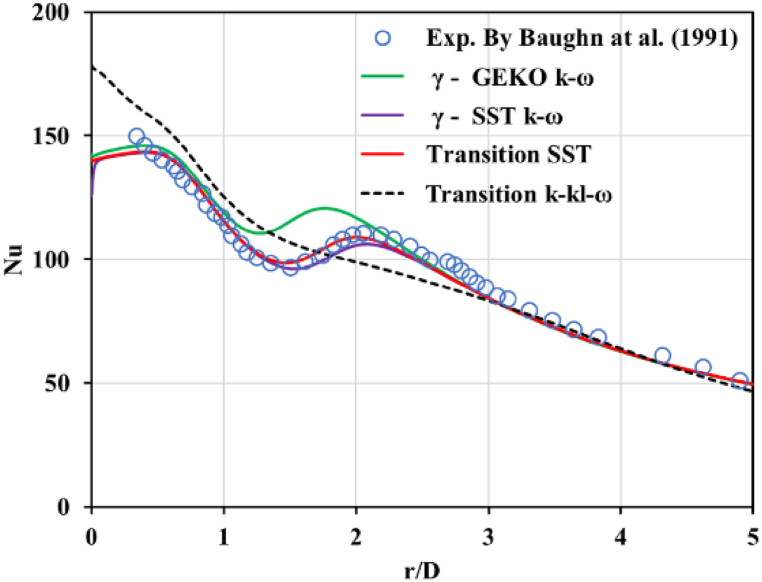
Comparisons between predicted and measured mean Nusselt number versus radial distance for different turbulence models.

To compare the four different turbulence models and their ability to predict relaminarization, the local averaged Nusselt numbers shown in Fig. 4 reveal that a stagnation zone (r/D < 0.1) formed directly under the jet where it impacted the surface. This stationary flow accelerated and after being laminar initially, it transitioned and became turbulent. The initial acceleration and associated convection caused a high convection heat transfer zone. Additionally, as the flow was laminar, no mixing occurred, and the heat transfer rate dropped at increasing radii. After the transition, the turbulent flow with its associated mixing enhanced heat transfer, leading to another local maximum heat transfer zone. Clearly, the simulated results from the transition SST model were in excellent agreement with the measurement data. The near-wall treatment in terms of destruction and the relaminarization source term from the transition SST model could capture the second peak of the Nusselt number. The intermittency factor played an important role in the turbulent kinetic energy transport equation of the SST model.

### 4.2 Normalized mean impingement jet velocity

**Fig. 5:**
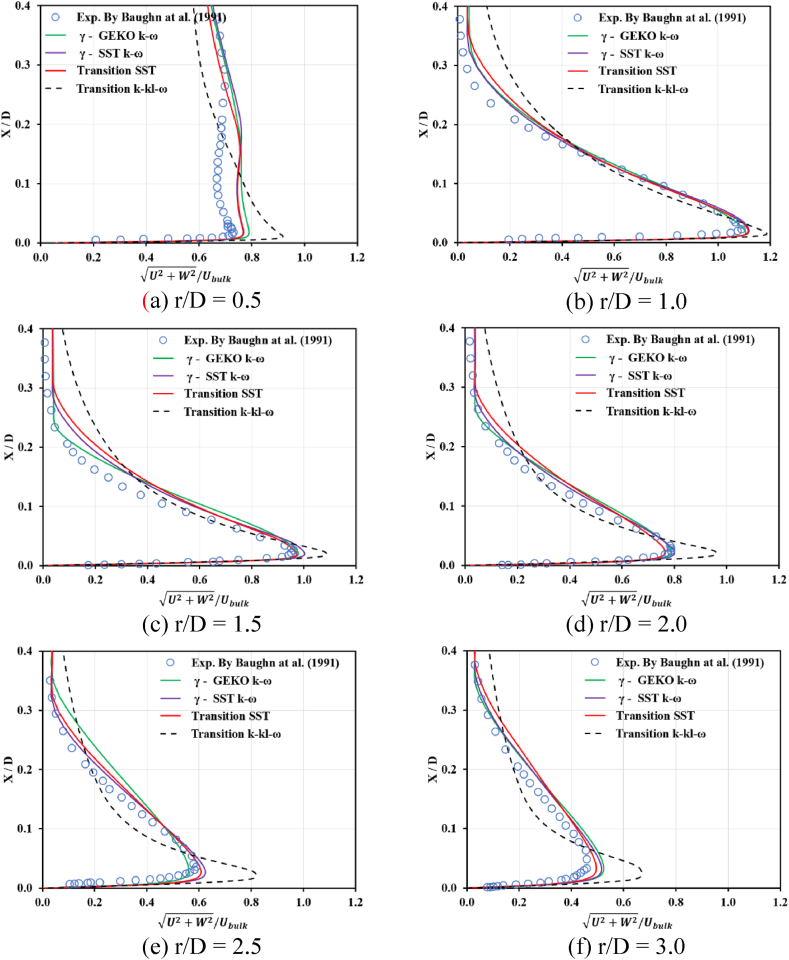
Predicted and measured normalized mean velocity versus vertical distance for different turbulence models.

As predicted by the transition SST model shown in Fig. 5 (a)-(f), the simulated and measured normalized mean axial and radial velocity components are demonstrated at radial positions r/D = 0.5, 1.0, and 2.5. The data from Cooper et al. [41] are included and compared. Based on these results, the collapse of turbulence was examined, showing the flow became lamina-like in the transition region, 1 ≤ r/D ≤ 3. The simulated mean velocity profile was used to explain the relaminarization process.

• For r/D < 1, the simulated results from all transition models in the study over-predicted compared to the experimental data; there was also an important difference when x/D > 1, as shown in Fig. 5 (a). However, a small discrepancy can be observed for γ−SST−k−ω and the transition SST results.
• Fig. 5 (b)–(f) shows the excellent agreement between the mean velocity profile predictions of the transition SST model and the experimental results for the transition region, 1 ≤ r/D ≤ 3, because additional destruction/relaminarization source terms are included in Eq. (1) to capture those transition flow mechanisms better during simulations. However, a greater discrepancy was found in the near-wall region for the interval r/D ≥ 2.0 as shown in Fig. 5 (d)–(f), requiring some improvement in the transition SST model prediction.
• The prediction results from transition k−kl−ω models produced the maximum error compared to the other transition models in the current study.

### 4.3 Normalized mean impingement jet turbulent kinetic energy

The turbulent kinetic energy is another main factor in transition models of jet impingement applications. The predicted turbulent kinetic energy results from the transition SST would be best compared with those of the studied transition models. More details in each region are summarized below.

**Fig. 6:**
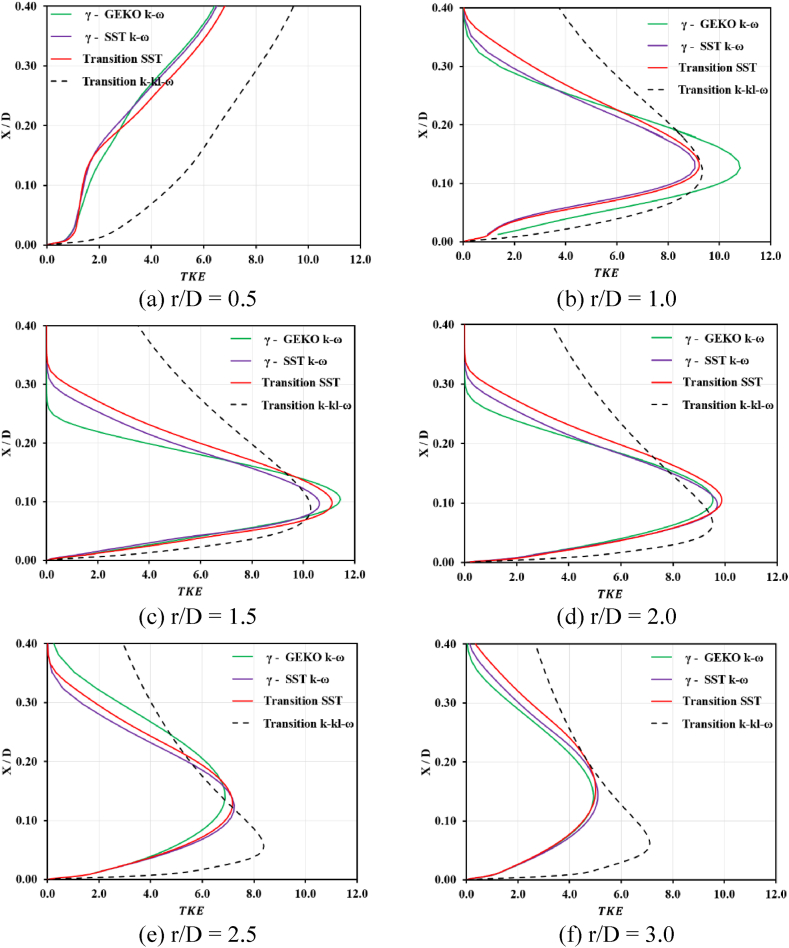
Predicted and measured normalized mean TKE versus vertical distance for different turbulence models.
• For 1 ≤ r/D ≤ 3, observation in this region revealed that the turbulent kinetic energy simulations from γ−GEKO−k−ω, γ−SST−k−ω, and transition SST were very similar, except for r/D = 1 in Fig. 6 (b), where the results from γ−GEKO−k−ω produced the maximum deviation from the transition SST models, as shown in Fig. 6 (e)–(f). As expected, the region of the secondary peak of the local Nusselt number was also over-predicted by the γ−GEKO−k−ω models compared with the experimental data presented in Fig. 4. The set of parameters used in the γ−GEKO−k−ω model may be adjusted to improve the accuracy for impinging jet applications.

• For r/D < 1, the simulated turbulent kinetic energy was almost similar, except for the transition k−kl−ω models. Over-prediction and different profiles can be seen in Fig. 6 (a).

### 4.4 Flow field and local surface nusselt number

**Fig. 7:**
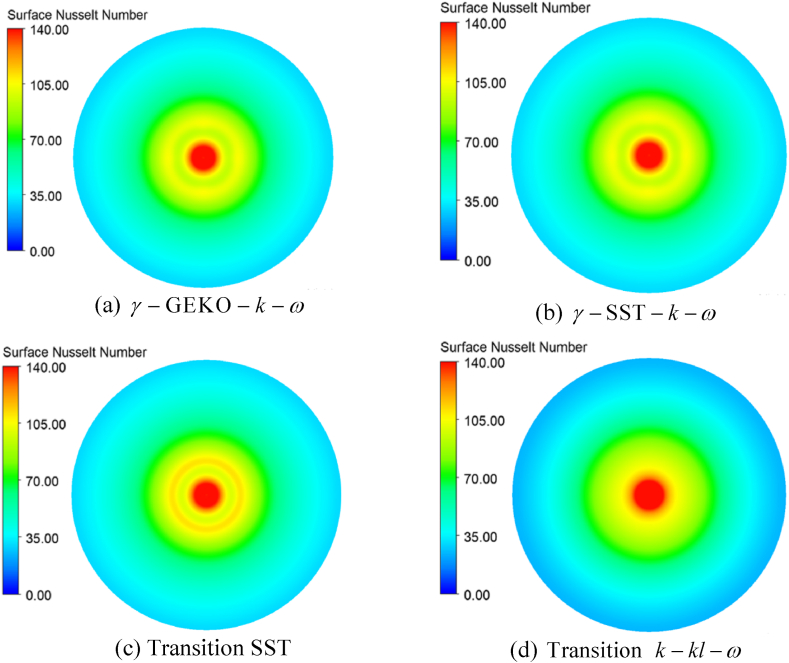
Local surface Nusselt number for different models.

**Fig. 8:**
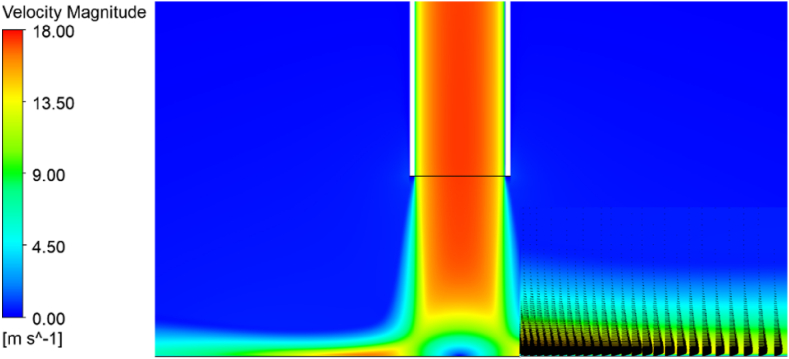
Local velocity vector for the transition SST model.

The local information of a jet impinging on the constant heat flux target is shown in Fig. 7 (a)-(d), which compares the contour plots of the local surface Nusselt number acquired from all transition models. The transition SST model was able to capture the second peak, illustrated by the two different red circular areas. Additionally, when we explore the flow field by velocity magnitude in Fig. 8, the velocity distribution near the wall had a parabolic profile, confirming that the near-wall region was vital to transfer the turbulence quantity from the inner region to the outer layer and then, when the flow quantity follows the correlation of separation-induced transition correction, relaminarization or re-transition will arise. This finding agreed with several studies, such as Aliaga et al. [43], Suzen and Huang [44,45], and Shah et al. [50]. Hence, this finding supports the implementation of these numerical results and their adaptability in our next research project or any relevant research project in the industry, although we only have limited computational resources.

## 5 Archeological applications

**Fig. 9:**
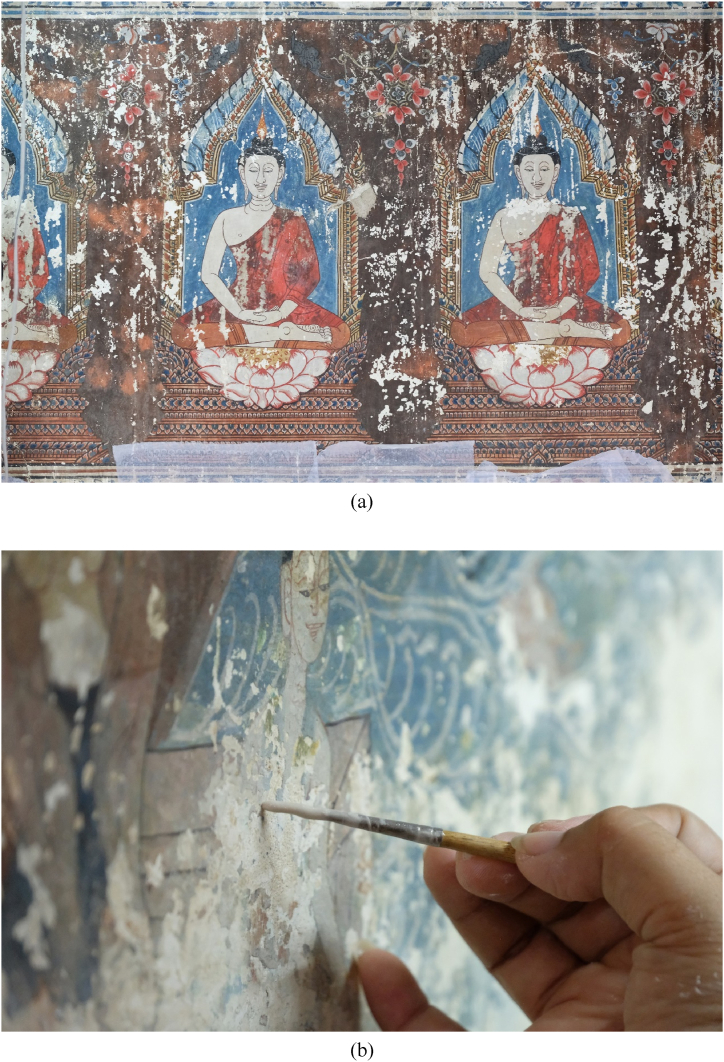
Ancient mural painting, (a) damage before restoration and (b) mural painting restoration using white clay filler.

From the previous section, we conclude that the transition SST turbulence model has the best accuracy in predicting the Nusselt number for the relaminarization of jet impingement on a flat plate. This section adapts it to archeological applications involving cleaning or restoration. Fig. 9 (a)-(b) describes the deterioration and restoration process using white clay filler with a high moisture content after restoration by an archeologist at Chan temple (Wat Chan) located in Si Prachan district, Suphanburi, province, Thailand.

**Fig. 10:**
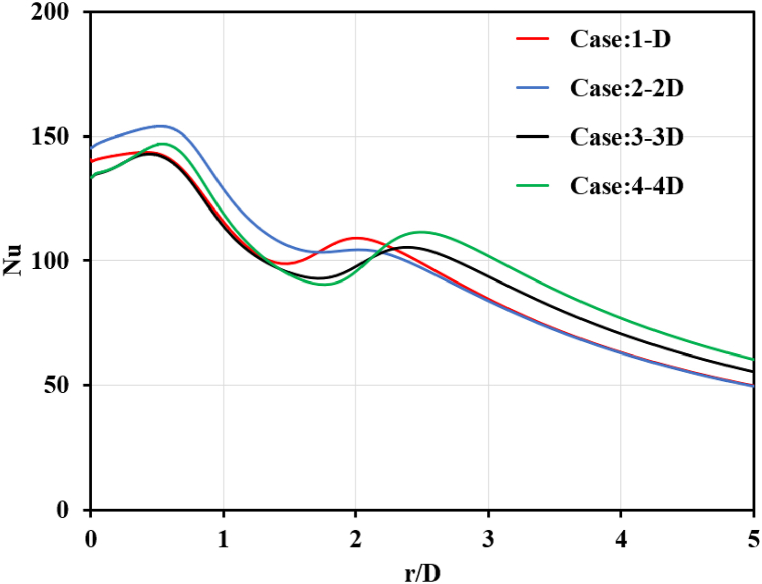
Nusselt number on wall with jet diameter variation.

To apply the jet impingement concept on a flat plate, hot air was used to slowly remove the water content in the white clay filler. It also helped to reduce initiating micro-cracks on the mural painting. We used the preliminary standard procedure to maintain Re = 23,000, representing 15.54 m/s of exit air velocity from the jet nozzle. After varying the jet diameters (D, 2D, 3D and 4D), the results confirmed that the second peak of the Nusselt number was still aligned on the golden ratio with 1 ≤ r/D ≤ 3. However, Nusselt number variation was evident because Re did not exactly equal 23,000. The interpretation is that no matter how much we change the value of r/D, it does not affect the second peak of the Nusselt number, as shown in Fig. 10. Notably, the critical shear force causes for delamination or peeling have not yet been reported and will require additional research.

**Fig. 11:**
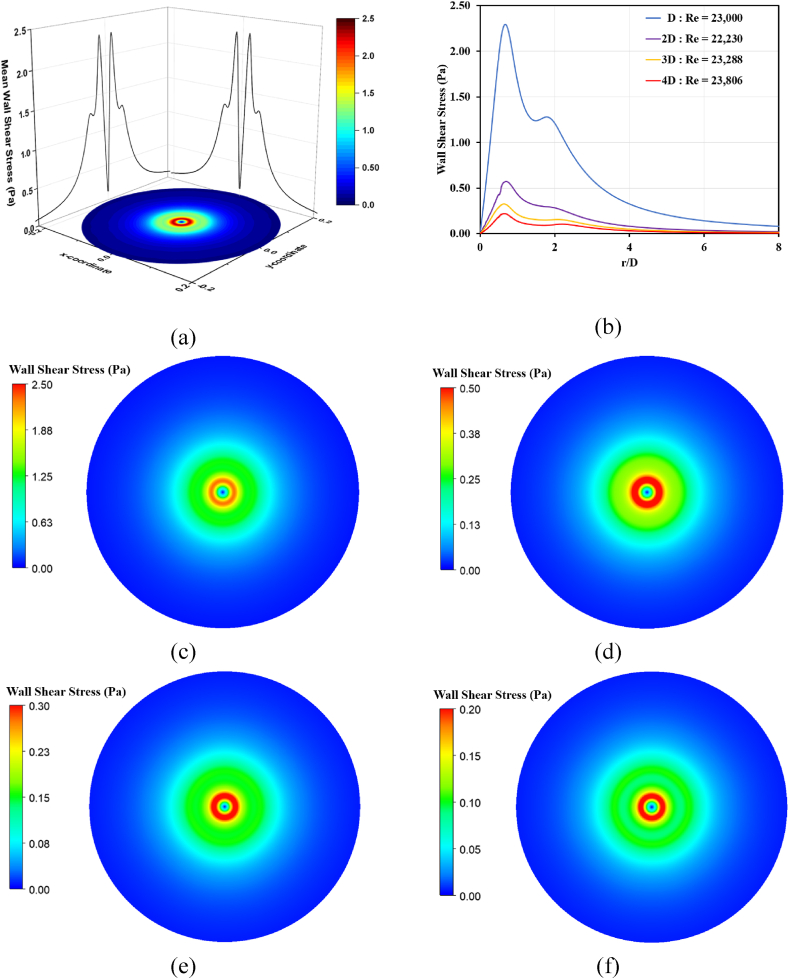
Wall shear stress distribution on wall; (a) 3D wall profile of wall shear stress, (b) The right half of wall shear stress distribution, (c) Re = 23,000, (d) Re = 22,207, (e) Re = 23,228 and (f) Re = 23,806.

Dehumidifying design equipment is often used in the conservation of ancient mural paintings to control the relative humidity and temperature of the environment in which the paintings are stored or displayed. The purpose of this equipment is to prevent the expansion and contraction of the paint layers due to changes in humidity which can cause cracking and flaking of the paint. It is important to have specific and sensitive dehumidifying design equipment for ancient mural paintings. Such equipment must be able to accurately control the humidity and temperature to minimize the risk of damage to the painting. Additionally, the equipment should be designed to exert minimal shear force on the mural wall. As we mentioned, Fig. 11 (a)-(f) shows that all cases of wall shear stress were homogeneous in all directions, meaning that the force exerted on the mural wall is distributed evenly in all directions. This is an important consideration in the equipment design, as it helps minimize the risk of damage to the painting by ensuring that the force exerted on the mural wall is as evenly distributed as possible. However, it is essential to note that no matter how sensitive the equipment is, it can never eliminate the risk of damage to an ancient mural painting. It is essential that the equipment is used in conjunction with other conservation techniques and that the painting is regularly inspected and maintained to ensure its preservation for future generations.

To address a concern of archeologist, heat transfer techniques can be used in the conservation of ancient mural paintings to remove dirt, grime, and other types of surface contaminants. This is usually done by heating the surface of the painting and then using a gentle stream of air or water to remove the contaminants. The heat can also help soften and loosen old varnish and over-painting, making the removal of the contaminants easier. This process is called “thermo-ablation” or “thermo-cleaning.” Hot-air or hot-water jet cleaning techniques are well known, as they reduce the amount of water and detergent needed and allow cleaning the surface with less mechanical action, avoiding damage to the painted surface. It is important to note that heat transfer techniques should be used with great care, as too much heat or pressure can damage the painting. Therefore, it is usually carried out by trained conservators who have experience in using these techniques.

## 6 Conclusion

The focus of the study was the validation of transition flow model which works with jet impingement applications. Reynolds averaged Navier Stokes (RANS) techniques, including a variation of transition options, such as γ−GEKO−k−ω, γ−SST−k−ω, transition SST and transition k−kl−ω, were used to simulate the turbulent flow fields. ANSYS-Fluent 2022R2 was the tool used in the study and a constant surface heat flux on a flat plate was chosen for the test case. The flow characteristics and averaged Nusselt number prediction were simulated and compared with other reported results. The following conclusions were drawn:

• The collapse of turbulence shows the flow became lamina-like in the transition region, 1 ≤ r/D ≤ 3. Also, the shape of velocity distribution within the same region is parabolic profiles near a target wall. It would confirm that the relaminarization mechanism is captured by the transition SST model in the research.
• For a given Reynolds number, the highest values of the predicted Nusselt number were located near the stagnation point and decreased monotonically within the wall jet regions.
• The γ−GEKO−k−ω and k−kl−ω transition models failed to predict the correct trend of the instantaneous computed Nusselt number distribution.
• The simulated results from the γ−SST−k−ω and transition SST with the destruction/relaminarization model provided strong agreement with experimental data.
• Secondary peaks of the Nusselt number in the radial direction may occur due to flow transition from being laminar to turbulent in the wall jet, as was apparent in the γ−GEKO−k−ω, γ−SST−k−ω, and transition SST models. In particular, the simulated results from the γ−GEKO−k−ω model over-predicted the second peak compared with the experimental data. Improvement of the γ−GEKO−k−ω model may be possible by adjusting the constant coefficient based on some turbulent parameter.
• The flow field was in good agreement with several other studies which supported the use and adaptation of the current models for relevant research projects both academic and industrial, although the authors have limited computational resources.
• Improvement is possible in all turbulence models (such as γ−GEKO−k−ω) according to this turbulence model design for generalized flow; however, this will require optimizing the model parameters to be consistent with specific flow applications.
• The second peak of the Nusselt number was still aligned on the golden ratio (1 ≤ r/D ≤ 3) which was not impacted by jet diameter variation. This range will be used in the next step of developing a prototype of a dehumidifying unit for use in archeological applications.
• Further research is required to extend the current application in archeology, such as how the cleaning process induces mural deterioration, refining the jet dehumidifying procedure on ancient material, and minimizing micro-cracking due to thermo-ablation or thermo-cleaning.

## CRediT authorship contribution statement

**Mongkol Kaewbumrung:** Conceptualization, Formal analysis, Writing – original draft, Writing – review & editing. **Chalermpol Plengsa-Ard:** Conceptualization, Formal analysis, Resources, Writing – review & editing.

## Declaration of competing interest

The authors declare that they have no known competing financial interests or personal relationships that could have appeared to influence the work reported in this paper.
